# Future research perspectives on environment and health: the requirement for a more expansive concept of translational cancer research

**DOI:** 10.1186/1476-069X-10-S1-S15

**Published:** 2011-04-05

**Authors:** Christopher P Wild

**Affiliations:** 1 International Agency for Research on Cancer, 150 cours Albert Thomas, 69000 Lyon cedex 08, France

## Abstract

The last two decades have seen exciting advances in understanding the human genome, aided by the development of powerful analytical laboratory tools. These advances have enabled genome-wide association studies to link specific genetic variants with an altered risk of cancer. Unfortunately there has not been an analogous refinement of tools on such a comprehensive scale to permit an equally thorough investigation of environmental factors, yet it is known that these play a major role in cancer etiology. This limitation led to the suggested need for an exposome to match the genome. Major advances both in understanding mechanisms of carcinogenesis as well as in the technology to investigate these underlying steps in the disease process offer the potential to redress this imbalance between exposome and genome. This is all the more important in order to fully exploit the large prospective cohort studies with biological specimens now being established to investigate the environmental and genetic basis of common chronic diseases. Currently translational cancer research is understood to equate to a “bench to bedside” process, focused on improved clinical management of cancer. Unfortunately, alone, this is an inadequate response to the growing burden of cancer worldwide. Priority also needs to be placed on understanding the causes of cancer, its prevention and, critically, how to implement promising interventions into health care structures. The need therefore is to translate basic science to the population in parallel to the translation into the clinic. This “two-way” translational cancer research encourages the common soil of basic science to be applied both to the prevention of cancer and to its treatment. In this way the notable advances in relation to carcinogenesis will yield a richer benefit to society through balanced initiatives to understand the causes and prevention of cancer in addition to more effective treatment and care of those people developing the disease.

## Introduction

The last two decades have yielded unprecedented understanding of the human genome and subsequently progress has been made in identifying associations between genetic variation and cancer risk [1,2]. This has been achieved partly through the development of an exquisite set of tools that permit a thorough characterization of an individual’s genetic make-up in relation to their phenotype. Genome-wide association studies based on a multicentre international design are therefore providing some novel insights into the genetic variants and associated biological pathways linked to cancer development and response to cancer therapy.

At the same time it is pertinent to remember the critical role of environmental (i.e. non-genetic) risk factors in the etiology of cancer [3]. The majority of cancers have a complex causation with a number of environmental factors acting on an individual’s genetic background to influence risk. However, the elucidation of these complex etiologies is often hampered by the inability to measure environmental exposures with the degree of precision or scope now available for genetic analyses. This limitation led to the suggested need for an exposome to match the genome [4,5].

The exposome is the comprehensive characterization of an individual’s lifetime exposure history, analogous to sequencing the 3 billion base pair genetic readout of the genome. It can be thought of as the summation of a sequential set of cross-sectional measurements that would build to a composite picture of lifetime exposure. The scope of the exposome is, by definition, broad, including lifestyle, nutrition, occupation, obesity and physical activity, environmental chemicals, infections, radiation etc. The exposome is also dynamic, varying over time both qualitatively (in composition) and quantitatively (in the level of a given component). Notwithstanding the daunting task to characterize the exposome of an individual, addressing it is now imaginable through major advances both in understanding mechanisms of carcinogenesis as well as through the technology available to investigate these same underlying molecular, biochemical and metabolic steps in the disease process [6,7]. Notably, it is increasingly evident that environmental agents may exert their carcinogenic effects through many diverse pathways, not only through somatic mutation but also epigenetic changes such as methylation, histone modification, miRNA etc [8]. It is the more recent comprehension of these latter pathways and the availability of tools to assess their modulation that opens such interesting new ways to consider the impact of the environment, in its broadest sense, on the risk of cancer development.

Continued failure to redress the current imbalance between the characterization of the exposome and the genome would have serious implications for cancer prevention. For example, major long-term investment has and continues to be made to establish large-scale prospective cohort studies that comprise not only complex questionnaire and clinical data but also extensive banks of biological material [9]. Without improvements in the tools to measure exposure it is unlikely that the prospective cohorts will yield their full promise to elucidate the environmental and genetic components of chronic disease as an evidence-base for prevention. It is imperative therefore that investment is made now to refine a set of tools to better investigate the environmental risk factors for cancer so that these are available a decade from now when the cohorts become mature in terms of the number of cases of disease occurring among the participants.

Biomarkers offer great promise in refining exposure assessment, establishing the biological plausibility of an exposure-disease association and in evaluating intervention studies [5]. Biomarkers do not represent the only opportunities for advance. For example, refinements in personal and environmental monitoring, geographic information systems, and increasingly sophisticated questionnaires will provide complementary approaches in the coming years. Nevertheless, the technologies of genomics, transcriptomics, proteomics and metabolomics offer new ways to investigate in epidemiological studies the biological pathways relevant to carcinogenesis [10]. In addition, developments in mass spectrometry are permitting, for example, the more comprehensive analysis of DNA adducts as biomarkers of exposure [7]. These biomarkers can be detected in the types of biological samples (plasma, serum, white blood cells) collected in epidemiological studies and stored in associated biobanks. The different tools therefore hold great promise for exposure assessment given the accumulating evidence that a given exposure can leave traces of its occurrence in terms of a particular pattern of gene expression, proteins or metabolites, although little is yet known about the duration of such alterations, the dose-response or the influence of confounding factors, for example [5].

Translational cancer research is generally understood to equate to a “bench to bedside” process. In that context advances in both the knowledge of carcinogenic mechanisms and the tools to interrogate these processes are targeted at improving the detection, diagnosis, treatment and prognosis of cancer. The goal of personalized medicine in particular is to use this refined information about the patient and their tumour in order to adapt the clinical management to the individual [11]. Examples of treatment regimes tailored to novel biomarker profiles in the tumour have already been reported [12]. The era of personalized medicine therefore provides a tremendous opportunity for academic researchers, clinicians and industry to collaborate in order to develop the cancer treatments of the future. The organizations funding cancer research also recognize the importance of support to this research area, where donors often are motivated by the understandable desire to “find a cure” for cancer.

The above approach, however, has its limitations when considered in a broader perspective. The burden of cancer worldwide is projected to rise dramatically in the coming 20 years, with an estimated 12.7 million new cases in 2008 set to rise to 21.4 million by 2030 based on demographic changes alone [13]. This represents a 69% increase in the annual number of new cancers in just over twenty years. For cancer deaths the projected increase is 72%, with approximately 13.2 million deaths in 2030 compared to 7.6 million in 2008 (Table 1). Furthermore this burden is not evenly shared. Whilst population aging will result in increases in cancer burden in the high income countries, already over half the new cancer cases (56%) and cancer deaths (63%) occur in the developing regions of the world. The disparity is set to rise further, based only on a combination of population growth and aging in these latter regions. If the underlying incidence of cancer increases concomitantly, due to more widespread tobacco use or a more westernized lifestyle for example, then the rise and inequality will be still more dramatic.

**Table 1:** Estimated global cancer burden in 2008 and projections to 2030 (values in millions)

	More developed		Less developed		World	
	2008	2030	2008	2030	2008	2030

**New cases**	5.6	7.4	7.1	12.9	12.7	21.4
**Deaths**	2.8	3.9	4.8	9.1	7.6	13.2

Consequently the greatest burden of new cancer cases will fall on some of the regions of the world least able to face the challenge. Improved treatment and care are important in reducing the suffering associated with this disease and it is therefore imperative that such services are developed [14]. Unfortunately, alone, this is an inadequate response. Priority also needs to be placed on understanding the causes of cancer, its prevention and critically, how to implement promising interventions into health care structures.

Many risk factors and prevention strategies are already established and in these cases implementation is the priority (e.g. reduced tobacco use; vaccination against hepatitis B virus and human papilloma viruses; increased physical activity; reduced obesity; screening for cervical, breast and colorectal cancers). However, much remains to be understood about the complex causes of cancer and how to intervene. These questions can be addressed by using the laboratory tools mentioned above to explore how risk factors act through genetic and epigenetic pathways in the development of cancer. This new generation of biomarkers may provide better approaches to measure exposure and provide evidence about the biological plausibility of an exposure-disease association. In addition, identification of the important pathways in human pathogenesis in relation to these exposures may yield evidence-based opportunities to modulate the course of disease development.

**Figure 1:**
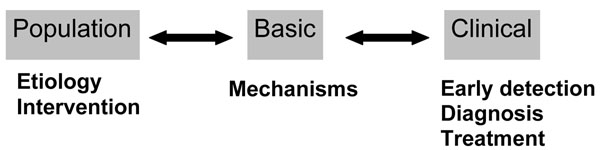
Two-way translational cancer research

Despite the above-mentioned advances in laboratory sciences there is a risk that the complementary opportunity for cancer prevention will not be fully realized unless the knowledge of mechanisms and the associated technology are driven towards applied research questions in the fields of epidemiology and public health. The need is to translate basic science to the population in analogous fashion to the translation into the clinic. Hence the call for “two-way” translational cancer research [15], where the common soil of basic science is applied both to cancer prevention and its treatment (Figure 1). It is surely this more balanced strategy, supported by appropriate investment, which will best equip a global response to the challenges of human cancer burden in the next two decades.

Will the opportunities of two-way translational research be seized? This will depend on a number of factors some of which are highlighted here. First, there is a need for inter-disciplinary research and the development of a new generation of bilingual researchers comfortable with the languages of laboratory and population sciences. This requires training but also structures within and across research organizations which facilitate and place value on such collaboration. Second, policymakers and funders must prioritise translational research into the causes and prevention of cancer on a scale not yet seen by comparison with translational research from bench to beside. Molecular cancer epidemiology requires a long-term investment of sufficient magnitude to permit the incorporation of biomarker development, validation and application into large-scale studies. This will in turn require recognition that for the commercial sector, investment in this type of research, particularly primary prevention, is generally less attractive and therefore public and charitable funding should be directed to compensate for this relative lack of market-value. International organizations such as the International Agency for Research on Cancer must provide an exemplar in this regard. The longer-term benefits of prevention in reducing the economic cost of cancer will be an eventual counterbalance to this investment. Third, policymakers need to be further sensitized to the limitations of improved clinical management of cancer as a response to the growing burden of the disease globally. Partly this requires increased support to cancer registries to provide more complete data on the current and future scale of the disease at a country and regional level. Cancer prevention and control policies need to be prioritised nationally. Fourth, the wider cancer community needs to find more effective ways to communicate the benefits of prevention research to the public so that the priorities of governmental and non-governmental organizations are influenced in this direction by the societies they serve.

In summary, translational cancer research stands at an exciting but critical point in time. The causes of a majority of cancers remain unknown, limiting the evidence-base for prevention. Advances in laboratory sciences offer clear opportunities to unravel the complex etiology of the disease, addressing both genetic and environmental risk factors notably through improved exposure assessment and information on mechanisms which can add persuasive evidence to the causality of exposure-disease associations. Major investments mean that large-scale cohort studies will provide the framework in which to apply many of these new tools. There is nevertheless a period of a decade or so before the cohorts mature in terms of numbers of cases, during which time progress can be made in the development and validation of the putative new biomarkers. This challenge would be best addressed by an internationally coordinated effort where the development of methods aimed towards a set of “priority exposures”, are addressed in an integrated way. If this were achieved the exposome would provide not only the complement to the genome but would mimic it in the collaborative approach required to bring the idea to fruition.

## Competing interests

The author declares that they have no competing financial or non-financial interests.
